# Subjective sleep quality and sleep habits of elderly inhabiting rural areas of Sambalpur district of Odisha, India

**DOI:** 10.1371/journal.pone.0314770

**Published:** 2024-12-05

**Authors:** Sarojini Minz, Monalisa Mohapatra, Uma Charan Pati, Pritipadma Sahu, Raghunath Satpathy, Rupashree Brahma Kumari, Pradosh Kumar Acharya, Nirupama Sahoo, Sujit Kumar Jally

**Affiliations:** 1 Center of Excellence, Odisha Center for Geriatrics and Gerontology, Gangadhar Meher University, Sambalpur, Odisha, India; 2 School of Chemistry, Gangadhar Meher University, Sambalpur, Odisha, India; 3 School of Economics, Gangadhar Meher University, Sambalpur, Odisha, India; Maulana Azad Medical College, INDIA

## Abstract

Sleep is an important physiological process that is essential for human beings because it maintains the circadian rhythm appropriately. The sleep behavior in the older population of India has not been studied adequately. Further, there is no report on the sleep behavior of the elderly population of Odisha, India. Therefore, this study has been designed to examine the status of sleep quality in the elderly living in rural areas of the Sambalpur district of western Odisha, India. This study includes1992 elderly subjects above the age of 60 years (945 females and 1047 males) who participated voluntarily. Data collection was done using a socio-demographic form and Pittsburgh Sleep Quality Index (PSQI) inventory to determine the sleep quality and related factors that might alter the quality of sleep. The prevalence rate of sleep quality was assessed for both groups and the independence of attributes was tested statistically using the Chi-square test with a*p-*value ≤0.05 was considered significant. Of the 1992 elderly participants, 1384 (69.5%) showed good sleep quality, and 608 (30.5%) had poor sleep quality. The factors, “female gender, marital status such as married” and “drinking habits of alcohol” are the statistically significant associations with good sleep quality among the elderly participants. The overall prevalence of good-quality sleep was high among the elderly participants living in rural areas of the Sambalpur district of western Odisha. These results might serve as a baseline database for future research endeavors. Further, a longitudinal study that has been planned might help in identifying the underlying factors that sustain good-quality sleep in a majority of the studied population.

## Introduction

Getting older is a natural, biological, and universal process for every living being on Earth. Needless to mention, population aging has become a serious concern the world over, physically, socially, psychologically, and epidemiologically too [1, 2]. Owing to a better quality of living and improved medical science and technology, life expectancy at birth has gone up significantly in recent years and as a consequence, the share of the older population is on the rise everywhere. The demographic structure of India’s population has changed significantly in the last decade or so. The share of the elderly population, i.e., 60 years and above has gone up from just 1% in the 1990s to around 10% at present and is expected to reach the 20% mark by mid of the current century. An international trend could be gauged if we refer to the Iranian census of 2006 which revealed that the proportion of people aged 60 or above in the country’s population was about 7.27%. By 2030, this rate is predicted to be between 25% and 30% [3, 4]. In addition to the increasing proportion of elderly in our total population, there are issues of concerns like the breaking down of the traditional joint family system and migration of youth from rural areas to urban or semi-urban centres for better job opportunities [5]. Older people are left behind in villages which is why their status is a cause of concerns for the policy makers. Many studies have proved that youth are withdrawing themselves from agriculture and preferring informal sector jobs in urban centres. The older segment of our population thus, is subjugated to socio-economic and psycho-cultural inhibiting factors and requires special attention and care due to their increasing morbidity and psychological issues of concern. If the already explored pieces of evidence are anything to be adhered to, then these indicate that aging is not only influenced by genes but also by behavioral factors, like sleep quality [6]. Sleep is an important physiological process that is essential for human beings because it maintains the circadian rhythm appropriately. The circadian clock which is situated in the hypothalamus of the human brain controls the sleep-wake cycle [7–9]. In 2013, Tel [10] reported that sleep is the best indicator of quality of life.

Sleep is essential for everyone because it’s required each day to restore energy for daily life. According to the recommendations of the National Sleep Foundation, elderly people should have an average sleep duration of 7–8 hours per day. Contrarily, insufficient sleep creates numerous difficulties in people including low quality of living and unhappiness. For example, people who sleep less than six hours a day have a 30% higher death rate than those who sleep seven to eight hours. More than 20% of people aged 60 years and above experienced a mental or neurological disorder and nearly 6.6% of all disabilities (disability-adjusted life years) were found among elderly people [11]. The two most prevalent disorders in these age groups are dementia and depression, which affect 5% and 7% of the older population, respectively [11].

**Fig 1:**
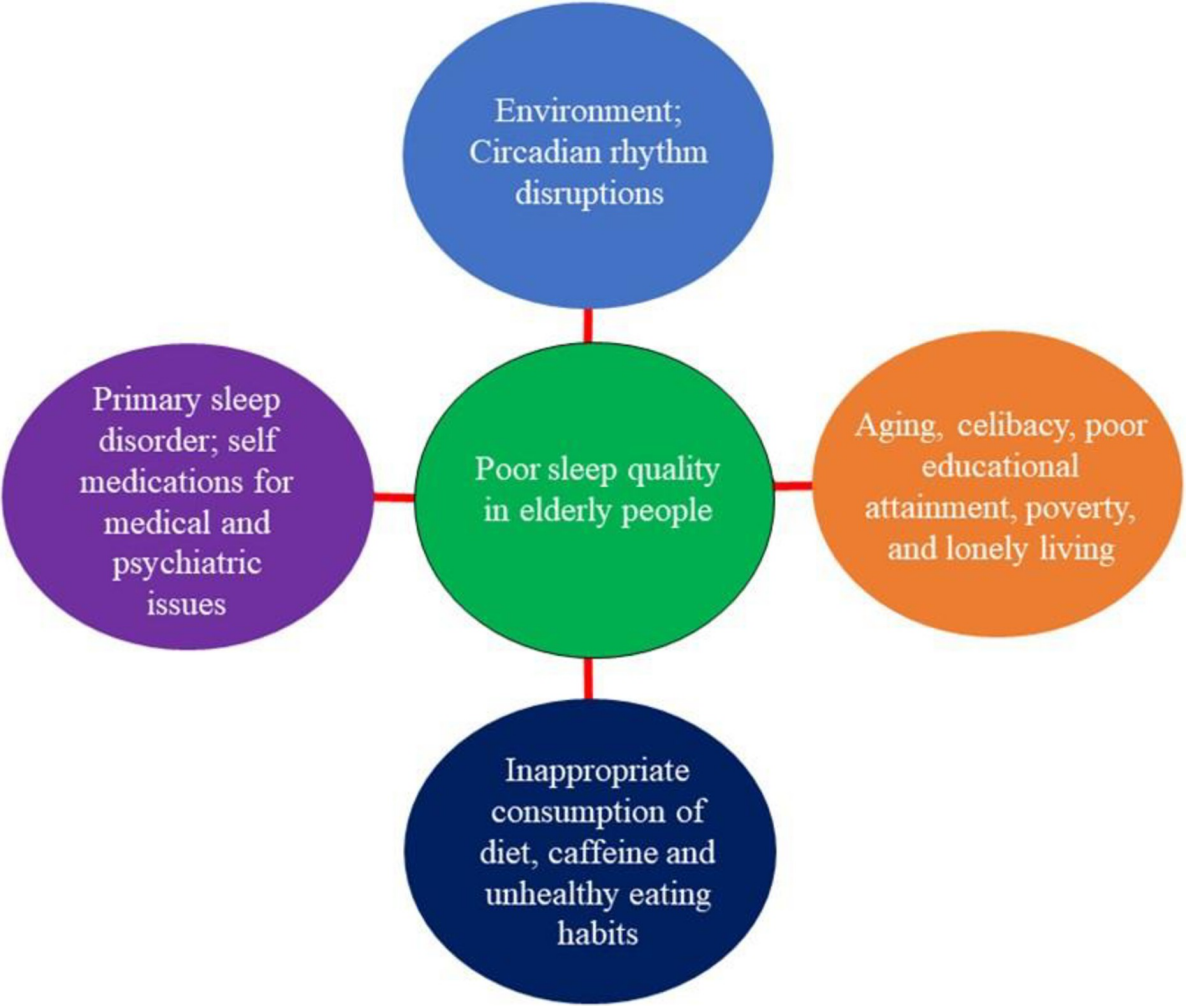
Factors responsible for poor sleep quality in elderly people.

Earlier studies have emphasized how the quality of life is strongly correlated with the quantity and quality of sleep [10, 12]. Energy, emotional stability, and health condition will eventually be affected by lack of proper sleep. Hence, sleep disorders, like insomnia and poor sleep quality can decrease the quality of life of people. Sleep disturbances and age have a high positive correlation and such disorders are commonly found in the elderly population due to various psychological and biological factors. The quality of sleep and quality of life declines with increasing age [10]. The prevalence of sleep complaints in older people may develop from primary endogenous age-related sleep problems. It might enhance disease occurrence and the use of medications, which may cause secondary sleep issues [13]. Poor sleep quality in individuals has many attributes that can be ascribed to factors like environment, pain, chronic diseases, and disturbance of sleep [14, 15]. The risk of developing heart disease, misery, falling, and accidents is associated with poor sleep quality. More or inadequate sleep among older individuals may be considered a marker of their poor quality of life and health status [16]. Incapability to sleep can harm the health of older adults in terms of consciousness issues, late response time, trouble in concentration, carelessness, falls, reduced overall performance in everyday lifestyle activities, severe depression, and anxiety [17]. Factors associated with poor sleep quality in elderly people have been illustrated in Fig 1.

There is no denying the fact that sleep requirement in human beings is a function of a multitude of factors, like the latitude they live in, their chronotype, gender, ethnicity, physiological state, and lifestyle. An in-depth review of the literature brings forth the fact that not enough studies have been conducted on the important issue of sleep behavior of the older population of India. Studies have not been carried out at the level of the state of Odisha as far as sleep behavior in the elderly population of the state is concerned. It is not only an interesting area of study, but it is also important to understand the socio-demographic and clinical characteristics of sleep problems, to minimize the negative effects that are caused by the poor quality of sleep. Therefore, the current study has been designed to examine the status of sleep quality in a population of elderly living in rural areas of the Sambalpur district of western Odisha, India.

## Materials and methods

### Participants from selected study areas

**Fig 2:**
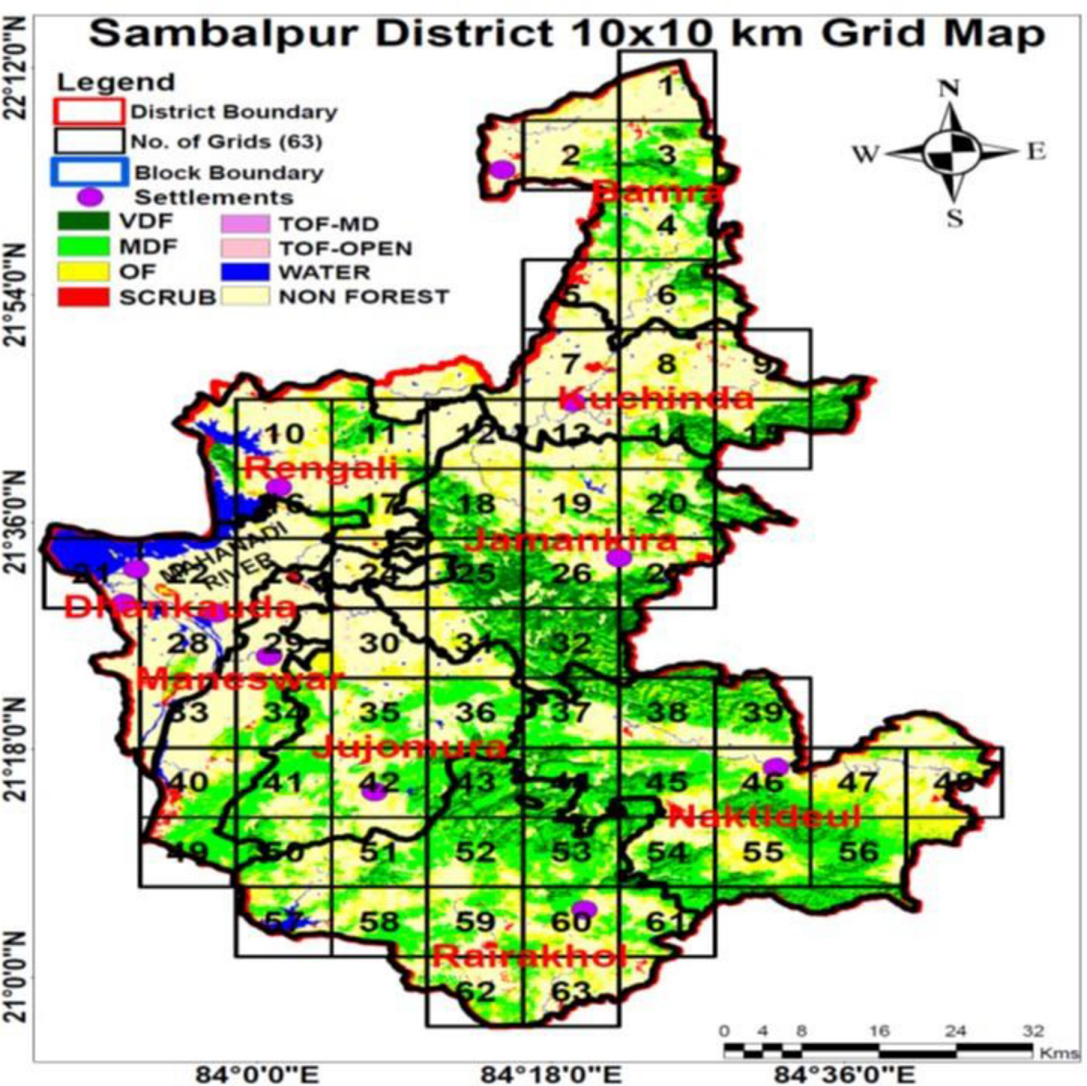
The grid map of Sambalpur district, Odisha, India.

This study includes 1992 elderly subjects above 60 years of age (945 females and 1047 males) from 65 villages of the Sambalpur district of Odisha who participated voluntarily and 10 subjects were excluded due to the incomplete questionnaire (details in Fig 2). For this work, data collection was done from participants belonging to Sambalpur district during the period from November 2022 to March 2023. Sambalpur is an old district situated in the western part of the state of Odisha. It is situated nearly 300 kms to the west of the state capital of Odisha i.e. Bhubaneswar. All villages on a district map were superimposed with 10 x 10 km^2^ grids with the help of experts (Fig 2). Fig 2 contains both shape files and Landsat-8 OLI satellite imagery. The shape files of villages, blocks and districts were obtained from the Survey of India. A 10 x 10 km^2^ grid was created using Arc Map 10.3 to facilitate the identification of suitable sampling sites based on the presence of human settlement. The freely available Landsat-8 image was obtained from the USGS Earth Explorer (earthexplorer.usgs.gov/) site and the forest cover analysis was prepared from the Landsat-8 OLI using Erdas Imagine 9.2 image processing software.

A systematic sampling method was adopted for the selection of the sample grids and the households within each selected grid were selected randomly and based on human habitation. After the identification of grids, 10 out of 63 grids were selected by the technique of systematic sampling. The selected grids were: 4, 10, 16, 22, 28, 34, 40, 46, 52, and 58.

**Fig 3:**
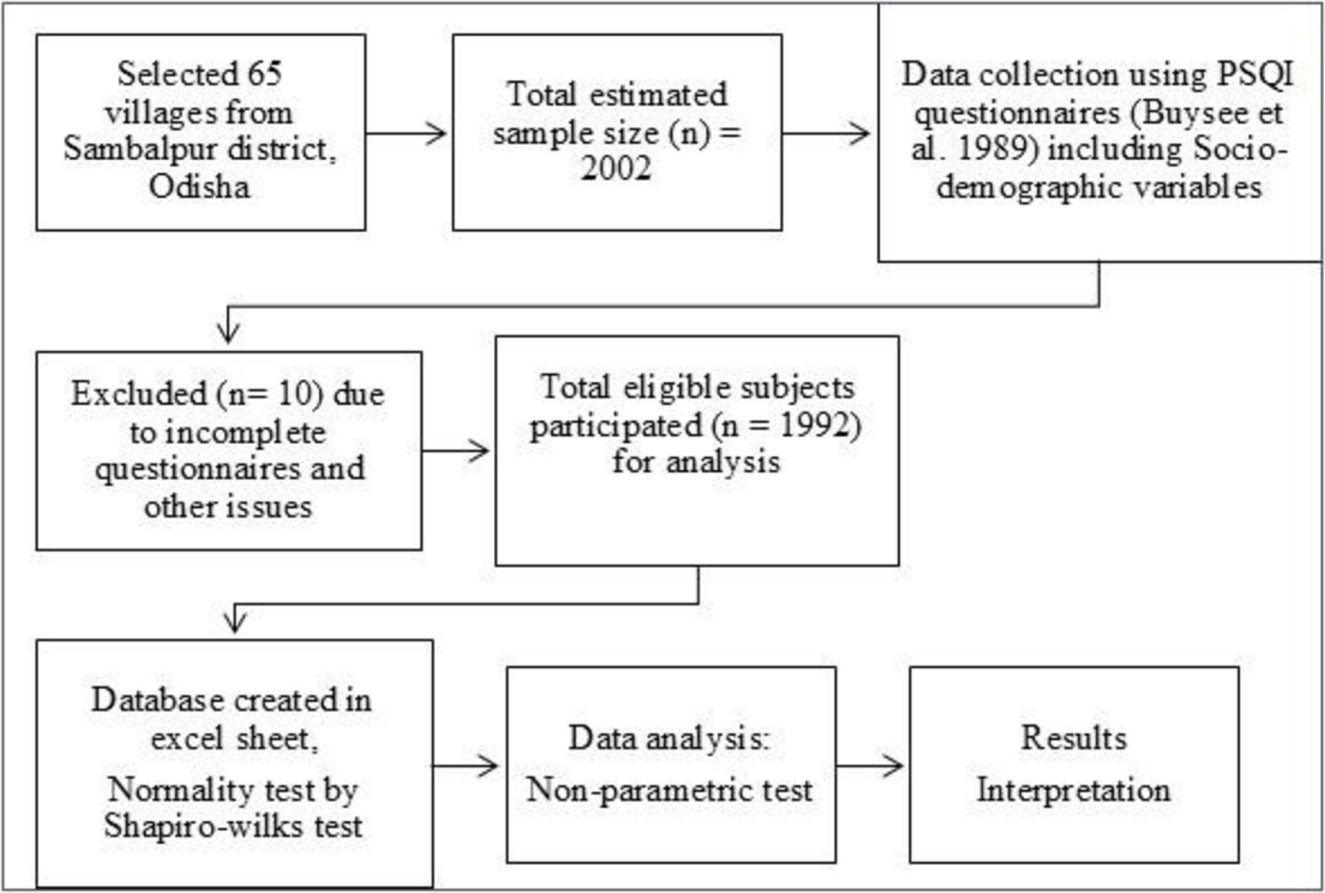
Flowchart of the procedure of selection of subjects and methodology for sleep quality study in elderly people of Sambalpur district of western Odisha, India.

Most of the subjects of this study were farmers, housewives, weavers, daily wagers, and retired persons. The total population of the Sambalpur district residing in rural areas is733,006 [18] (male:364314and female:368,692). A Pilot study was conducted before administering the schedule to gauge the reliability and consistency of primary data. Since subjective sleep quality is a universal problem/issue not confined to any particular groups or sections, our emphasis was on selecting data from all categories of people. The procedure of selection of subjects and methodology for sleep quality study in elderly people is discussed through a flowchart given in Fig 3. Participants were selected randomly from different socio-cultural and economic groups like farmers, weavers, daily wagers, professionals, housewives and retired persons. We have access to information that could identify individual participants during or after data collection. As far as the selection of Sambalpur district as the study district is concerned, we adopted the convenience sampling technique to collect primary data from different parts of rural pockets. A stratified random sampling technique was adopted to select participants from various sections of the population across groups residing in rural pockets of the district.

### Participant’s inclusion criteria

The individual elderly subjects who were 60 years and above, and provided valid informed verbal consent before starting the investigation were included in this study.

### Participant’s exclusion criteria

The elderly subjects with pre-existing severe health conditions, like paralysis, mental abnormality, speech and vision problems, below 60 years of age, and who did not agree to participate were excluded from this study. Those who filled out the questionnaires incompletely were removed from the database.

### Pittsburgh Sleep Quality Index (PSQI)

The PSQI is a self-reported questionnaire that assesses the sleep quality of an individual over the past month. This was developed by Buysse et al. 1989 [19]. It contains 19 items that measure different aspects of sleep including components like sleep quality, sleep latency, sleep duration, habitual sleep efficiency, sleep disturbance, use of sleeping medication, and daytime dysfunction. Scores on these seven components are used to compute a global score reflecting an individual’s sleep quality [19–21]. Each component of the scale ranges from 0–3 [22]. The global PSQI score was calculated by totaling the scores of seven components in a Microsoft (MS) Access Database Application. The global PSQI provides an overall rating ranging from 0–21 [20, 21], where a score of ≤5 denotes a healthier sleep quality, and any score >5 denotes poor sleep quality.

### Approval of the study design and protocol from IEC for human research

The study design and protocol were ethically approved by the Institutional Ethics Committee (IEC) for human research at Gangadhar Meher University, Amruta Vihar, Sambalpur, Odisha, India (IEC ref no.9858). This study was conducted following relevant guidelines and regulations. We explained the importance of the study to each subject before including them as a part of the data collection process. The verbal consent of the subjects was taken before the investigation, and that has also been approved by the ethical committee. Privacy and convenience of the subjects were given utmost importance before including them as a part of the study and also during the time of data collection.

### Data collection/study method adopted

The aim of the study was explained to each participant before soliciting their responses at the beginning of the data collection work. After that, the subjects filled out the demographical data sheet. The demographical data sheet gathered information on age, gender, height, weight, smoking, and drinking habits, and the health status of the subjects. We used PSQI to determine the sleep quality of elderly subjects inhabiting rural areas of the Sambalpur district of western Odisha. The individual elderly subjects were interviewed face-to-face and their responses were recorded by the field investigators.

### Data analysis

A database in the Excel Worksheet was created for the application of statistical techniques. Descriptive statistical methods were used for PSQI global score, and its 7 components using the SPSS 16.0 and Excel data analysis Tool Pack. The normality and homogeneity of the data were checked using the Shapiro-Wilk test. Table 1 represents the Normality test of the global PSQI score and seven components of elderly people of rural areas of the Sambalpur district. Assumptions of the normality test and homogeneity of variance across the studied groups were violated by the data on sleep quality and all components (Shapiro-Wilk statistic, *p*<0.05). Therefore, the non-parametric tests were applied to analyse PSQI scores. The prevalence rate of sleep quality was assessed for both groups and the independence of attributes was tested statistically by the Chi-square test. The *p-*value ≤0.05 was considered significant. The differences in sleep quality in different age groups with gender were tested using the Kruskal-Wallis test at the significance level *p*<0.05 and the Mann-Whitney U test was used for pair-wise comparisons between different age groups. Further, Bonferroni correction was used, and the significance level was <0.008;/<0.01, i.e., (0.05/6). The impact of socio-demographic variables on sleep quality was assessed by using a binomial logistic regression model. The binomial logistic regression analysis was done by using the Software Jamovi 2.5.6.

**Table 1:** Normality test of global PSQI score and seven components of elderly subjects (N = 1992) of rural areas of Sambalpur district.

Components	Skewness	Kurtosis	Shapiro-Wilk Statistic	*p*-value
	Z = static/SE	Z = static/SE		
Sleep quality	20.146	-2.1	0.707	<0.001
Sleep latency	-2.855	-2.064	0.841	<0.001
Sleep duration	33.182	22.555	0.570	<0.001
Habitual sleep efficiency	133.382	588.609	0.152	<0.001
Sleep disturbances	-4.527	19.136	0.612	<0.001
Sleep medication	99.982	286.7	0.208	<0.001
Day dysfunction	25.364	5.536	0.631	<0.001
Global PSQI scores	12.964	5.891	0.946	<0.001

## Results

The mean age of the participants was 69.71 (±8.01 SD) years. The data given in Table 2 depicts the socio-demographic characteristics that include age, gender, marital status, education, and health status of the elderly population of rural areas of Sambalpur district.

**Table 2:** Socio-demographic characteristics of elderly subjects (N = 1992) who participated voluntarily in the study.

Socio-demographic variables	Frequency	Percentage (%)
Gender		
Male	1047	52.6
Female	945	47.4
Age (Years)		
60–69 (male = 540, female = 560)	1100	55.2
70–79 (male = 353, female = 222)	575	28.9
80–89 (male = 129, female = 143)	272	13.7
≥90 (male = 25, female = 20)	45	2.3
Marital status		
Married	1509	75.8
Widowed	478	24.0
Unmarried	5	0.3
Education		
Illiterate	1391	69.8
Primary (1–7)	449	22.5
Secondary (8–10)	135	6.8
Higher Secondary (11-12/Graduate)	17	0.9
Chronic Disease		
Yes	814	40.9
No	1178	59.1
Sleep habits	Average time	
Bedtime (h:min)	21:03	
Wake time (h:min)	5:15	
Sleep duration (h)	Mean	SD
	7.92	1.02

### Distribution of participants based on components of the Pittsburgh Sleep Quality Index

The data given in Table 3 represents the distribution of frequency of seven components of PSQI. It reported that the responses of participants, 57.5% of the elderly had very good and 14.3% very bad subjective sleep quality;11.3% had a sleep latency of >60 minutes; and 0.4% of the elderly had a sleep duration of <5 hours. Habitual Sleep Efficiency was >85% for 97.0% of the subjects. Only 5% of the elderly subjects had used some sleep medication during the last month and 1.9% had a very difficult level of daytime dysfunction. The overall score was good i.e., ≤5 in 69.5% of elderly participants, whereas it was poor i.e.,>5 in just 30.5% of study participants.

**Table 3:** Distribution of participants (N = 1992) based on components of the Pittsburgh Sleep Quality Index.

Components of PSQI	Female	Male	Frequency/Percentage
Sleep quality			
Very good	499 (25.1)	647 (32.5)	1146 (57.5)
Fairly good	192 (9.6)	221 (11.1)	413 (20.7)
Fairly bad	80 (4.0)	68 (3.4)	148 (7.4)
Very bad	174 (8.7)	111 (5.6)	285 (14.3)
Sleep latency (in minutes)			
<15	40 (2.0)	56 (2.8)	96 (4.8)
16–30	285 (14.3)	379 (19.0)	664 (33.3)
31–60	494 (24.8)	513 (25.8)	1007 (50.6)
>60	126 (6.3)	99 (5.0)	225 (11.3)
Sleep duration (in hours)			
≥7	686 (34.4)	814 (40.9)	1500 (75.3)
>6-<7	192 (9.6)	161 (8.1)	353 (17.7)
5–6	64 (3.2)	68 (3.4)	132 (6.6)
<5	3 (0.2)	4 (0.2)	7(0.4)
Habitual sleep efficiency			
>85%	918 (46.1)	1014 (50.9)	1932 (97.0)
75–84%	22 (1.1)	29 (1.5)	51 (2.5)
65–74%	3 (0.2)	4 (0.2)	7 (0.4)
<65%	2 (0.1)	0 (0)	2 (0.1)
Sleep disturbances			
Not in the last month	96 (4.8)	179 (9.0)	275 (13.8)
Once a week	781 (39.2)	807 (40.5)	1588 (79.7)
1–2 times a week	67 (3.4)	60 (3.0)	127 (6.4)
More than thrice a week	1 (0.1)	0 (0)	2 (0.1)
Use of sleep medication			
Not during the past month	898 (45.1)	996 (50.0)	1894 (95.1)
Less than once a week	25 (1.3)	25 (1.3)	50 (2.5)
Once or twice a week	13 (0.7)	14 (0.7)	27 (1.4)
Three or more times a week	9 (0.5)	12 (0.6)	21 (1.1)
Daytime Dysfunction			
Not difficult	665 (33.4)	726 (36.4)	1391 (69.8)
Little difficult	137 (6.9)	130 (6.5)	267 (13.4)
Difficult	126 (6.3)	171 (8.6)	297 (14.9)
Very difficult	17 (0.9)	20 (1.0)	37 (1.9)
Global PSQI score			
≤ 5	617 (31.0)	767 (38.6)	1384 (69.5)
> 5	328 (16.5)	280 (14.0)	608 (30.5)

### Distribution of subjective sleep quality based on age and gender

Out of the 1992 elderly participants, 69.5% showed good sleep quality, and 30.5% had poor sleep quality. In this study, 65.3% of females and 73.3% of males reported ≤5 PSQI scores, whereas34.7% of females and 26.7% of males showed poor sleep quality with PSQI scores >5. The mean score (± 1 SD) for good sleep quality varied as a function of gender. It was 3.15 ± 1.26 in females and 2.96± 1.30 in males, and for poor sleep quality, the values were 7.25± 1.61 for females and 7.22± 1.47 for males (Table 4).

**Table 4:** Gender and age-wise distribution of sleep quality in elderly participants (N = 1992) of rural areas of Sambalpur district.

Characteristics/Attributes	Good (≤5)	Poor (>5)	*χ*^2^-value(df)	*p*-value
	N (%)	N (%)		
Gender				
Female (945)	617 (65.3)	328 (34.7)	14.863 (1)	<0.001
	(3.15 ± 1.26)^§^	(7.25 ± 1.61)		
Male (1047)	767 (73.3)	280 (26.7)		
	(2.96± 1.30)	(7.22 ± 1.47)		
Age groups (years)				
60–69 (1100)	792 (72.0)	308 (28.0)	32.877 (3)	<0.001
	(2.96± 1.36)	(7.29± 1.53)		
70–79 (575)	414 (72.0)	161 (28.0)		
	(3.19± 1.16)	(7.34± 1.55)		
80–89 (272)	156 (57.4)	116 (42.6)		
	(3.12± 1.24)	(6.94± 1.49)		
≥90 (45)	22 (48.9)	23 (51.1)		
	(2.95± 1.13)	(7.30± 1.89)		
Educational Level				
Illeterate	959 (68.9)	432 (31.1)	0.912 (3)	0.822
(1391)	(3.00± 1.27)	(7.21± 1.50)		
Primary (1–7)	315 (70.2)	134 (29.8)		
(449)	(3.07± 1.39)	(7.40± 1.74)		
Secondary (8–10)	98 (72.6)	37 (27.4)		
(135)	(3.35± 1.12)	(6.89± 1.15)		
Higher (+2 & +3)	12 (70.6)	5 (29.4)		
(17)	(3.58± 0.90)	(8.00± 2.00)		
Having a Bed Partner				
Having(1509)	1126 (74.6)	383 (25.4)	77.669 (2)	<0.001
	(2.95± 1.31)	(7.21± 1.48)		
Lost(478)	255 (53.3)	223 (46.7)		
	(3.45± 1.09)	(7.25± 1.62)		
Not Having(5)	3 (60.0)	2 (40.0)		
	(4.00 ± 1.00)	(9.50 ± 4.95)		

^§^(Mean ± SD) (*p*<0.05)

Based on age groups, out of the total subjects, 72.0% belonged to the 60–69, and 70–79 age groups, and57.4%, and 48.9%, to 80–89, and ≥90 age groups, respectively, had good sleep quality and few people exhibited poor sleep quality, irrespective of age groups (Table 4). The mean (± 1 SD) for good sleep quality was found to be 2.96 ± 1.36, 3.19 ± 1.16, 3.12 ± 1.24, and 2.95 ± 1.13, and, the values were 7.29 ± 1.53, 7.34 ± 1.55, 6.94 ± 1.49 and 7.30 ± 1.89for poor sleep quality of different elderly age groups, respectively (Table 4). The result of the Chi-square test showed a statistically significant association between sleep quality and gender/age among the elderly subjects at the level *p*<0.001(Table 4).

### Distribution of subjective sleep quality based on level of education and the status of having a bed partner or not

Based on the level of education, good sleep quality was reported by 68.9%of illiterate, 70.2% primary, 72.6%secondary, and 70.6% of highly qualified elderly subjects, and fewer participants exhibited poor sleep quality. The mean (± 1 SD) for good sleep quality was found to be 3.00 ± 1.27, 3.07 ± 1.39, 3.35 ± 1.12, and 3.58 ± 0.90, and for poor sleep quality, they were 7.21 ± 1.50, 7.40 ± 1.74, 6.89 ± 1.15 and 8.00 ± 2.00 in elderly participants, respectively (Table 4).

Sleep quality can be seen in terms of whether a person is having a bed partner or not. It is found from the analysis of data that 74.6% of respondents have a bed partner, 53.3% have lost his/her bed partner, and 60.0% of elderly respondents without having a bed partner reported ≤5 PSQI scores, whereas the same category of respondents reporting poor sleep quality (with PSQI scores >5) respectively have been 25.4%, 46.7% and 40.0%. The mean score (± 1 SD) for good sleep quality varied as a function of the status of having a bed partner or not. It was 2.95 ± 1.31for respondents having a bed partner,3.45± 1.09for those who have lost his/her bed partner, and 4.00 ± 1.00 for respondents without a bed partner and for poor sleep quality, the values were 7.21± 1.48,7.25 ± 1.62, and 9.50 ± 4.95 for the three categories of elderly respondents respectively (Table 4).

The result of the Chi-square test did not exhibit a statistically significant relationship between ‘sleep quality’ and ‘level of education,’ whereas a statistically significant (*p*<0.001) relationship between ‘sleep quality’ and ‘the status of having a bed partner or not’ was found among the elderly participants living in the rural pockets of the study area (Table 4).

### Variation of subjective sleep quality by age groups with gender

Table 5 represents personal sleep variables (mean ± 1 SD) that include seven components of the PSQI and the global PSQI score, respectively, of elderly participants inhabiting rural areas as a function of gender and age categories. The global PSQI scores by age groups of females ranged from 4.42 ± 2.35 to 5.40 ± 3.30, whereas in males, it ranged from 3.92 ± 2.43 to 5.00 ± 2.14. The results of the Kruskal-Wallis test exhibited a statistically significant effect on two components i.e. sleep quality and sleep disturbances only for females under different age categories (Table 6), whereas for males, a statistically significant effect was found for five components i.e., sleep quality, sleep latency, sleep duration, sleep disturbances, daytime dysfunction and global PSQI score (*p*<0.05). Furthermore, when different age groups were compared pairwise by the Mann-Whitney U test, it was revealed that the sleep quality of the 60–69 y age groups was statistically significantly (*p*<0.01, Bonferroni adjustment) different from other age groups, namely 80–89 y, and the sleep disturbances of 60–69 y showed statistically significantly varies from ≥90 y of female elderly participants. However, in the case of elderly males, one pair (60–69 vs 80–89) age groups found statistically significant in sleep duration, and two pairs of comparisons were exhibited statistical significance in sleep quality, sleep latency, daytime dysfunction, and global PSQI score. Moreover, five pairs of comparisons showed statistically significant differences in sleep disturbances of male elderly participants of rural areas of the Sambalpur district (Table 6).

**Table 5:** Subjective sleep quality (mean ± 1 SD) of elderly participants (N = 1992) inhabiting the rural areas of the Sambalpur district and comparison thereof.

Components	60–69 (y) (n = 560)	70–79 (y) (n = 222)	80–89 (y) (n = 143)	≥90 (y) (n = 20)	Kruskal Wallis test	
Female (945)					χ2-value (df)	p*-* value
Sleep quality	0.80 ± 1.10*	1.01 ± 1.18	1.27 ± 1.27*	1.05 ± 1.19	19.413 (3)	<0.001 ^**a**^
Sleep latency	1.72± 0.72	1.81 ± 0.71	1.74 ± 0.79	1.90 ± 0.97	2.806 (3)	0.422
Sleep duration	0.39 ± 0.63	0.32 ± 0.61	0.27 ± 0.58	0.20 ± 0.41	8.586 (3)	0.035
HSE	0.03 ± 0.20	0.05 ± 0.24	0.05 ± 0.32	0.05 ± 0.22	3.491 (3)	0.322
Sleep disturbances	0.95 ± 0.41*	1.00 ± 0.41	0.97 ± 0.44	1.25 ± 0.44*	12.007 (3)	0.007 ^**a**^
Sleep medication	0.09 ± 0.45	0.08 ± 0.37	0.04 ± 0.20	0.10 ± 0.45	0.314 (3)	0.957
Day time dysfunction	0.44 ± 0.76	0.54 ± 0.88	0.38 ± 0.71	0.85 ± 0.99	6.000 (3)	0.112
Global PSQI Score	4.42 ± 2.35	4.81 ± 2.45	4.73 ± 2.34	5.40 ± 3.30	6.006 (3)	0.111
Male (1047)	60–69 (y) (n = 540)	70–79 (y) (n = 353)	80–89 (y) (n = 129)	≥90 (y) (n = 25)		
Sleep quality	0.61 ± 0.98*	0.61 ± 0.97	0.95 ± 1.09*	1.00 ± 1.04	20.995 (3)	<0.001 ^**a**^
Sleep latency	1.54 ± 0.73*	1.68 ± 0.67*	1.86 ± 0.80*	1.56 ± 0.77	24.082 (3)	<0.001 ^**a**^
Sleep duration	0.35 ± 0.64*	0.27 ± 0.58	0.19 ± 0.50*	0.12 ± 0.33	11.332 (3)	0.010 ^**a**^
HSE	0.03 ± 0.19	0.04 ± 0.22	0.05 ± 0.25	0.00 ± 0.00	1.041 (3)	0.791
Sleep disturbances	0.81 ± 0.50*	0.93 ± 0.40**	1.01 ± 0.42*	1.28 ± 0.46**	42.489 (3)	<0.001 ^**a**^
Sleep medication	0.08 ± 0.41	0.08 ± 0.40	0.12 ± 0.52	0.00 ± 0.00	2.600 (3)	0.458
Day time dysfunction	0.50 ± 0.83*	0.45 ± 0.79*	0.59 ± 0.86	1.04 ± 1.02**	12.551 (3)	0.006 ^**a**^
Global PSQI Score	3.92 ± 2.43*	4.06 ± 2.09*	4.77 ± 2.33**	5.00 ± 2.14	20.328 (3)	<0.001^**a**^

^HSE^Habitual sleep efficiency

^a^<0.05, after Bonferroni correction Mann-Whitney test p-value

*<0.008/0.01 is significant

**Table 6:** Binomial Logistic regression coefficients for PSQI score by socio-demographic variables in a cohort consisting of rural elderly subjects (N = 1992) of Sambalpur district.

Variables	N	Binomial Logistic regression analysis		
		Odd ratio	95% of Confidence Intervals	p-value
Male gender	1047	0.73	0.58–0.92	0.01
Married Marital status	1509	0.45	0.36–0.58	<0.001
Literate	601	1.19	0.95–1.51	0.13
Country liquor consumption	186	1.80	1.28–2.54	<0.001
Alcohol consumption	1263	0.56	0.35–0.90	0.02
Smoker	70	1.05	0.59–1.89	0.86
Tobacco consumption	295	0.82	0.59–1.14	0.25
Tea/coffee consumption	1913	1.17	0.95–1.45	0.14

### Regression models for socio-demographic variables predicting sleep quality

Effects of socio-demographic variables and drinking habits on the sleep quality of elderly subjects from rural areas were evaluated using a binomial logistic regression model. The overall regression was found to be statistically significant *p*<0.001. Factors like “gender, the status of having a bed partner or not”, education level and “drinking habits of country liquor and alcohol” are the statistically significant determinants of sleep quality among the elderly participants of rural areas of Sambalpur district, Odisha (Table 6).

The result of binomial logistic regression analysis showed that being male (compared to female) decreases the log odds of good sleep quality by 0.3141, or decreases the odds by approximately 27%. Being unmarried (compared to married) decreases the log odds of good sleep quality by 0.7877, or decreases the odds by approximately 55%. Having higher education (compared to lower education) decreases the log odds of good sleep quality by 0.1777, but this result is not statistically significant (p = 0.133). Not consuming alcohol (compared to consuming alcohol) decreases the log odds of good sleep quality by 0.5790, or decreases the odds by approximately 44%. The consumption of tobacco, tea/coffee and smoking have estimates that are not statistically significant (p > 0.05), suggesting no strong evidence of their effect on sleep quality in this model (Table 6).

## Discussion

Having good quality sleep and also of an adequate duration is of utmost importance to human beings and most specifically to the elderly segment of our population has already been examined in this research effort. This section presents a detailed discussion with necessary statistical evidence on the sleep quality of the elderly inhabiting the rural areas of the Sambalpur district of western Odisha.

In this study, more than half of the elderly (69.5%) showed good sleep quality which aligns with the previous result, that more than half of the elderly (65.2%) had shown good sleep quality [23]. Contrarily, this result did not support the earlier results that more than half of the elderly had experienced poor quality of sleep in a study examined in Dehradun (64.5%) [24], in Kerala (72.0%) among elderly residing in rural areas [17], 73.5% in elderly people living in a Nursing Home in Damghan City [25], 76.8% had ≥5 scores in elderly living in a private elderly care institution, Malaysia [14], and 78.0% in Chinese elderly people [26]. However, poor sleep quality was lower (30.5%) in our study and 15.9–29.4% as showed in an earlier study conducted on elderly people residing in rural and urban areas of China and Iran [27–29] than in other studies conducted among elderly subjects (31–49.7%) from different countries [30–38]. Based on the responses of the participants, 73.5% elderly had very good subjective sleep quality and most of the elderly subjects were satisfied with their sleep. The bed time of rural elderly people on average was nearly 9.0 pm and the wake-up time reported was 5.0 am. The average sleep duration of these elderly participants was nearly 8 hours daily which is very close to the recommended sleep length for older adults by the National Sleep Foundation [39] and this value is higher than the same derived from earlier studies conducted in mainland China (7.5 hours) [40] and another in Hong Kong, China (5.51 ± 1.5 h/night) [26]. This variation might be because all the participants of this current study were Hindu, most of the elderly subjects were physically active and healthy and residing in noise-free environments of rural areas of western Odisha, India. It has been reported earlier that the rural Indians–mainly Hindus are morning active. Pati et al. [41] reported that 75% of Indians are morning active. Further, the report by Nag and Pradhan [42] supports the conjecture. They reported that about 99.6% of subjects inhabiting remote areas without electricity are morning active. The average sleep duration of these subjects was 8.95 hours. However, there are not many studies on the average sleep duration of the rural populace of India. Additionally, Indians also believe in the axiom, “early to bed and early to rise, makes a man healthy, wealthy, and wise,” pronounced by Benjamin Franklin. The Hindu religious traditions also favor the Brahma Muhurta–about one hour 36 minutes before sunrise as the right time for performing meditation and prayer. This cultural practice might have induced early rising behavior and reportedly good sleep quality among the elderly people of western Odisha, India.

In this study, the mean global PSQI score is 4.32 ± 2.37 which is determined as healthier sleep quality than the same concluded by earlier studies conducted in different countries that is >6.0/7.0 scores indicating poor sleep quality among elderly people. Earlier studies reported that a lower level of social support was associated with a higher risk of poor quality of sleep among older people in nursing homes [43–45]. As a result, improving social support for the elderly is an essential factor in preventing sleep problems. The difference in global PSQI score and sleep quality of elderly people residing in rural areas of western Odisha, India, and the same in other countries and other states like Kerala [17], and Dehradun [24] is astonishing. The global PSQI score varied in different countries, such as Taipei at 6.3 ± 4.40 [46], Turkey at 7.28 ± 3.97 [47], Malaysia at 7.1 ± 3.40 [15], China at 7.74 ± 3.06 [37], and China at 7.24 ± 3.25 [48], respectively. The different lifestyles, cultures, and geographic variations of people living in different nations may be the cause of this difference in the global PSQI scores. Other factors like exposure to artificial lights at night, exposure to sunlight during the daytime, work pressure, social commitments, social support, and family economic status might be influencing sleep quality among elderly people. But all these factors have not been given space in this particular study and future researchers should take up field work and explore the possible causal mechanisms involving all the above-mentioned factors.

Moreover, studies on the relationship between gender and quality of sleep produced contradictory results. According to clinical and pre-clinical research, biological sex and sex steroids affect sleep patterns and sleep disorders [49, 50]. Gender variation in insomnia may be due to differences in psychiatric morbidity prevalence, symptom endorsement, gonadal steroids, sociocultural factors, and coping mechanisms [51]. Since a higher proportion of females have lower socio-economic status and are more prone to anxiety and depression, numerous studies have shown that poor sleep quality is more prevalent in females than in males [25, 34, 52]. Contrarily, some studies indicate that males were more likely than females to have reported having trouble sleeping [53, 54]. In this current study, poor sleep quality is seen more in females than in males, and the result of the Chi-square test showed a statistically significant association between sleep quality and gender among elderly subjects, which is comparable with the findings of the previous studies. Bahrami et al. [25] reported that sleep quality is statistically significantly associated with gender, age, and marital status. Conversely, the study conducted in Turkey showed no statistically significant association between sleep quality and gender [34]. The same results were seen in primary care centers in Malaysia [54], and nursing homes in Taiwan [55]. Lack of exercise was the primary risk factor for poor sleep quality among females [56] and elderly women living alone experienced poorer sleep quality than the older men [57]. The variations in these results may be attributed to differences in the sample of the study, pattern of the study, and study area.

However, there was a statistically significant association between sleep quality and the status of having a bed partner or not, although, Aliabadi et al. [36] did not find a significant association between sleep quality and marital status. As compared to those who had lost their partner, the sleep quality was better in the case of people with a bed partner, maybe due to the strong family support they had. This outcome finds corroboration from earlier results [32, 33, 58]. Conversely, Chaudary et al. [24] reported that widowed persons reported better sleep quality than married people. In this case, the reason may be the conflicts in marriages that act as stressors.

As far as this study is concerned it is found only 5% of elderly participants had used any medication to induce sleep in the previous month in contrast to only 2.0% shown in the previous study conducted in Dehradun [24]. These results are in sharp contrast to 14.1% of Chinese elderly adults resorting to sleep-inducing medications in nursing homes [21] and46.0% in America [59]. If these outcomes are any indications, sleep deficiency issues in elderly people have not been given sufficient care/attention by nursing home managers and health care workers.

Unruh et al. [60] have reported, specifically age-related variability in variables, such as sleep time, sleep efficiency, and sleep stages among aged people (≥80-year-olds). In our study, the result of the Chi-square test showed a statistically in significant association between sleep quality and age groups among the elderly subjects. Further, the results of the Kruskal-Wallis test showed a statistically significant difference in two components only i.e. sleep quality and sleep disturbances for females under different age categories, and for males, a statistically significant effect was found in five components i.e., sleep quality, sleep latency, sleep duration, sleep disturbances, daytime dysfunction and global PSQI score (*p*<0.05). Information on PSQI sleep variables in elderly people residing in tropical and subtropical countries is limited. The results obtained in this study are therefore valuable and add new information to the existing knowledge base.

The results of the regression analysis revealed that factors like “gender, the status of having a bed partner or not, level of education” are statistically significant. Additionally, drinking habits of “country liquor, and alcohol” were also identified as statistically significant. Furthermore, the result of binomial logistic regression analysis showed that being male is linked to a lower likelihood of experiencing good sleep quality compared to being female. This suggests that, on average, females tend to report better sleep quality than males. being unmarried, compared to being married, is associated with a significant decrease in the likelihood (or odds) of experiencing good sleep quality. This suggests that marital status has an impact on sleep quality, with married individuals generally having better sleep quality compared to unmarried individuals. The avoiding alcohol is associated with a substantial decrease in the likelihood of having good sleep quality, highlighting a significant impact of alcohol consumption habits on sleep outcomes. However, the broad limitations of this study can’t be lost sight of before we draw valid conclusions. First, the current study has used PSQI to determine the sleep quality of elderly people, which might not be very objective in nature. Although objective measurement methods (physiological measurements such as Polysomnography) are desirable, they are not usually included in large-scale epidemiological studies in the general population. Therefore, self-reported and survey-based measures remain the most commonly used measures in community research [61]. Secondly, the scope of this study is devoid of determining the cause-and-effect relationship between poor sleep quality and the risk factors associated with it.

## Conclusions

In this study, good quality of sleep was found to be high among the elderly participants living in rural areas of the Sambalpur district of western Odisha. Factors like “female gender, the status of having a bed partner” and “drinking habits of country liquor/alcohol” are the statistically significant associations with good sleep quality among these elderly participants. These results may serve as a basis for future research and a longitudinal study that has been planned might help in identifying the underlying factors that sustain good-quality sleep in a majority of the studied population.
